# Subtherapeutic Kitasamycin Promoted Fat Accumulation in the Longissimus Dorsi Muscle in Growing–Finishing Pigs

**DOI:** 10.3390/ani14071057

**Published:** 2024-03-30

**Authors:** Ge Han, Jie Yu, Jun He, Ping Zheng, Xiangbing Mao, Bing Yu

**Affiliations:** Key Laboratory of Animal Disease-Resistant Nutrition and Feed of China Ministry of Agriculture and Rural Affairs, Institute of Animal Nutrition, Sichuan Agricultural University, Chengdu 611130, China; haggie0215@icloud.com (G.H.); yujie@sicau.edu.cn (J.Y.); hejun8067@sicau.edu.cn (J.H.); zpind05@163.com (P.Z.);

**Keywords:** kitasamycin, fat accumulation, cecal microflora, growing finishing pigs

## Abstract

**Simple Summary:**

Besides the well—known adverse effects of antibiotic usage such as drug resistance, residue, and immune system suppression, there is emerging evidence suggesting their potential to induce fat accumulation in the body, which can affect human health and the quality of animal products. Kitasamycin (KM) is widely used to treat various conditions like upper respiratory tract diseases, dysentery, and fibrosis. This study aimed to investigate the effects of different doses of KM on lipid metabolism using pigs as models. The pigs were fed diets containing varying levels of KM: none, subtherapeutic, and therapeutic doses. The findings revealed that the subtherapeutic KM diet resulted in fat accumulation in back fat thickness, plasma, and longissimus dorsi muscle accompanied by alterations in both cecal microflora and concentrations of short—chain fatty acids (SCFAs). Interestingly, the therapeutic KM diet had decreased intestinal weight and density accompanied by improved apparent digestibility of nutrients. Overall, this study underscores the dose-dependent impact of KM on intramuscular fat deposition and underscores concerns regarding the potential risks associated with subtherapeutic and therapeutic KM doses in both human and animal disease treatment.

**Abstract:**

Kitasamycin (KM), a broad—spectrum macrolide antibiotic, has implications for growth performance and residue in animals and humans. This study aimed to explore the effects of different KM doses on intramuscular fat accumulation, cecal microflora, and short—chain fatty acids (SCFAs) using a growing–finishing pig model. Forty—two pigs were divided into three groups: control, subtherapeutic KM (50 mg/kg, KM50), and therapeutic KM (200 mg/kg, KM200) diets over 8 weeks. KM50 led to increased back fat thickness, fat content in the longissimus dorsi muscle (LM), and elevated plasma total cholesterol (TC) levels (*p* < 0.05), supported by upregulated lipid synthesis gene expression (*Acc1*, *Fas*, *Scd1*) (*p* < 0.05) in the LM. KM50 altered cecal microflora, reducing *Lactobacillus* spp. and *Bifidobacterium* spp. abundance, while increasing SCFA concentrations (acetic acid, propionic acid, total SCFAs) (*p* < 0.05). KM200 had minimal effects on intestinal weight and density, with increased apparent digestibility of nutrients. These findings highlight the dose-dependent impact of KM on intramuscular fat deposition. Subtherapeutic KM induced ectopic fat deposition, emphasizing potential risks in disease treatment for humans and animals.

## 1. Introduction

Antibiotics are extensively utilized in humans and veterinary medicine to combat diseases and prevent pathogenic bacterial infections [1]. Nonetheless, concerns regarding adverse outcomes such as drug resistance, residue presence, and immune system inhibition underscore the need for reasonable antibiotic use, particularly in food due to the increasing demand for meat production [2,3,4]. Consequently, many countries have restricted or prohibited the use of antibiotics as growth promoters in livestock [4,5]. Furthermore, recent clinical and rodent research has suggested a potential link between antibiotics and obesity-related metabolic disorders, with specific antibiotics implicated in gut microbiota changes and lipid ectopic deposition [6,7,8,9,10]. The altered gut microbiota has been reported to play a key role in host metabolism, as indicated by the ability to replicate metabolic disorders in germ-free recipients inoculated with penicillin-altered bacteria [11]. However, the underlying mechanisms of antibiotics-induced metabolic changes through gut microbiota remain unknown.

Kitasamycin (KM), a macrolide antibiotic, has extensive applications in treating upper respiratory tract diseases and dysentery caused by Gram—positive bacteria, Mycoplasma, and Rickettsia in humans and animals [1,12]. Its inhibitory effects on protein synthesis make it valuable in various therapeutic contexts, including fibrosis prevention after fistulating glaucoma surgery [12]. In both weaned piglets and growing–finishing pigs, KM has been shown to inhibit diarrhea, but has adverse effects on intestinal barrier function [13,14]. However, there are limited studies exploring the impact of different doses of KM on the metabolic health of pigs.

Pigs not only serve as a valuable human biomedical model due to their anatomical and physiological similarities with humans [15], but also represent a significant commodity in the meat production industry. However, the impact of different KM levels on lipid metabolism in pigs remains incompletely elucidated. The longissimus dorsi muscle area (LM) in swine is crucial for assessing ectopic fat deposition [16], affecting feed efficiency and carcass quality. Furthermore, lipid metabolism is intricately connected with the gut microbiome, with certain antibiotics potentially influencing host energy metabolism and lipid levels through alterations in microbiome composition and short—chain fatty acids (SCFAs) [17,18]. Therefore, comprehending the impact of KM on the gut microbiome is essential to grasp how KM may affect lipid metabolism in pigs.

In this study, KM was administered at both subtherapeutic and therapeutic doses in the diet. Despite the prevalence of KM use, there is a paucity of literature examining its influence on growth performance, gut microbiome, and intramuscular lipid metabolism in Duroc × (Yorkshire × Landrace) (DLY) pigs. The objective of this study was to examine the impact of different KM doses on growth performance, gut microbiome, SCFAs, fat deposition, and gene expression associated with lipid metabolism. Our findings revealed dose-dependent effects of KM on LM fat accumulation, cecal microflora, and SCFAs in pigs, providing novel insights into the potential side effects of KM utilization in this context.

## 2. Materials and Methods

### 2.1. Ethics Approval

The Animal Care Advisory Committee of Sichuan Agricultural University (Ya’an, China, No. 20190129) approved all experimental procedures.

### 2.2. Animals and Experimental Design

Forty-two DLY pigs, averaging 63.32 ± 1.00 kg in weight, were randomly assigned to three groups. Each group comprised 7 replicates, with 2 pigs per replicate. Within each group, six replicates contained one castrated male and one female, while one replicate included two females. Pigs received three different KM diets: a control diet (basal diet, 0 mg/kg KM), a subtherapeutic KM diet (KM50, 50 mg/kg KM in basal diet), and a therapeutic KM diet (KM200, 200 mg/kg KM in basal diet). KM with a purity of 50% was procured from Hainachuan Company (Guangdong, China). Dosage levels were determined based on the available content of KM with diets formulated to meet or exceed nutrient requirements recommended by the National Research Council (NRC, 2012) [19] for pigs at different growth periods (Table 1).

### 2.3. Animal Housing and Sampling

Pigs were housed in a climate—controlled barn (25 °C, 65% relative humidity) with ad libitum access to water and feed. The 8-week experiment comprised three periods (Period 1: week 1–2; Period 2: week 3–5; Period 3: week 6–8). Fecal and dietary samples were collected four days before concluding the experiment for apparent total tract digestibility (ATTD) measurements.

At the end of the trial, one pig per pen was randomly selected for fasting blood sample collection from the precaval vein. Serum was obtained through centrifugation (3000× *g*, 10 min) and stored at −20 °C for subsequent analysis of total cholesterol (TC) and total triglyceride (TG). Following blood collection, pigs were electrically stunned and exsanguination. Hot carcass weight, used for dressing percentage calculation, was measured without blood, hair, hoofs, and viscera. Back—fat thickness was determined at the first rib, last rib, and last lumbar vertebrae. The LM was measured at the 10th rib on the left side and a 150 g, 1—cm—thick LM sample was collected and frozen in liquid nitrogen. Additionally, cecal digesta were collected and stored at −80 °C for subsequent analyses of bacteria abundances and microbial metabolite concentrations.

### 2.4. Intestinal Index

Relative intestinal length, density and weight were calculated as per Godwin et al. [20] with the formulas provided as follows:

Relative intestinal length (cm/g) = intestinal length/body weight.

Relative intestinal density (g/cm) = intestinal weight/intestinal length.

Relative intestinal weight (%) = intestinal weight/body weight × 100.

### 2.5. Chemical Analyses

Nutrient digestibility was evaluated through the analysis of samples obtained from both dietary intake and fecal excretion at the culmination of the animal study. Prior to analysis, these samples were subjected to drying at 55 °C in a forced-air drying oven and subsequently finely ground. The ATTD of nutrients was determined using acid—insoluble ash (AIA) as an indicator. AIA content in fecal and dietary samples was quantified according to the AOAC Method 950.49. Fecal and dietary samples were utilized for analyzing dry matter (Method 930.15), crude protein (Method 990.03), and ether extract (Method 945.16), following AOAC protocols [21]. Gross energy was measured using an oxygen bomb calorimeter (Parr Instrument Co., Moline, IL, USA). The ATTD of nutrients was calculated as (100 − A_1_F_2_/A_2_F_1_ × 100), where A_1_ represents the AIA content of the diet, A_2_ represents the AIA content of feces, F_1_ represents the nutrient content of the diet, and F_2_ represents the nutrient content of feces.

### 2.6. Biochemical Analyses

Plasma concentrations of TC and TG were determined using commercial kits obtained from Nanjing Jiancheng Bioengineering Institute (Nanjing, Jiangsu, China) and an automatic biochemistry analyzer (Hitachi, Ibaraki-ken, Japan).

Intramuscular fat content was accessed following AOAC Method 988.05 [21]. Approximately 40 g of LM samples were cut, freeze-dried, pulverized, and assessed for intramuscular fat content.

SCFAs were isolated and quantified from cecal digesta samples using a gas chromatographic system (VARIAN CP-3800, Palo Alto, CA, USA) following Chen et al.’s method [22]. Concentrations were calculated using the formula: C_(SCFAs)_ = A × 4 × 1.223 × V/W, where C represents the concentrations of SCFAs; A represents the gas chromatography measurement data, V represents the volume of ultrapure water, and W represents the weight of the cecal digesta sample.

### 2.7. Microbial Real—Time Quantitative PCR (RT—qPCR)

Bacterial DNA in cecal digesta was extracted using the EZNATM Stool DNA kit (Omega BioTek, Doraville, CA, USA). The abundance of total bacteria and specific strains (*Escherichia coli*, *Lactobacillus*, *Bifidobacterium*, and *Bacillus*) was assessed by RT—qPCR using SYBR Premix Ex Taq reagents (TaKaRa Biotechnology, Dalian, China) and PrimerScriptTM PCR kit (TaKaRa Biotechnology, Dalian, China), respectively. Standard curves were generated using standard plasmids based on Chen et al.’s work (2013) [23]. Specific primer sequences and probes for RT—qPCR are provided in Table 2.

### 2.8. RNA Extraction, Reverse Transcription, and RT—qPCR

Total RNA from LM was extracted, purified, and reverse—transcribed using Trizol reagent (TaKaRa Biotechnology, Dalian, China), Qiagen RNeasy Mini kit (Qiagen, Valencia, CA, USA), and PrimeScript TM RT reagent kit (TaKaRa Biotechnology, Dalian, China), respectively. RNA quality was determined by a spectrophotometer (Beckman Coulter Inc., Fullerton, CA, USA) at 260 and 280 nm, with OD260:OD280 ratios ranging from 1.8 to 2.0 in all samples.

Expression levels of target genes in LM were analyzed by RT—qPCR using SYBR Premix Ex Taq reagents (TaKaRa Biotechnology, Dalian, China) and CFX—96 RT—PCR Detection System (Bio—Rad Laboratories, Hercules, CA, USA). The reaction mixture comprised 5 μL of SYBR Premix Ex Taq TM II, 0.5 μL of forward primers (4 μM), 0.5 μL of reverse primers (4 μM), 3 μL of double—distilled water, and 1 μL of complementary DNA, resulting in a total volume of 10 μL. Cycling conditions were as follows: an initial denaturation step at 95 °C for 10 s, followed by 40 cycles of denaturation at 95 °C for 5 s, annealing at 60 °C for 25 s, and extension at 72 °C for 15 s. After amplification, a melting curve analysis was conducted to assess the specificity and purity of all PCR products. The standard curve for each gene was executed three times to ensure reliable amplification efficiency. The Pfaffl method [24] was applied for data analysis, with β—actin as the reference gene. Primer sequences are provided in Table 3.

### 2.9. Statistical Analysis

GraphPad Prism (Version 9.0; GraphPad Software, La Jolla, CA, USA) was used for data analysis. Normality was assessed using the Shapiro–Wilk test. One—way ANOVA, followed by Tukey’s post hoc multiple comparisons test, was applied to normally distributed data. Logarithmic transformation was performed for asymmetrically distributed data, with subsequent analysis using one–way ANOVA if transformed data exhibited normal distribution. If the transformed data did not display normal distribution, the Kruskal–Wallis test with the post hoc Dunn’s multiple comparisons test was used. Statistical significance was set at *p* < 0.05, and results were reported as mean ± standard error.

## 3. Results

### 3.1. The Impact of Different Doses of KM on Growth Performance and Apparent Total Tract Digestibility

At the commencement of the experiment, pigs across all groups exhibited similar weights (Table 4). During Period 1, subtherapeutic and therapeutic KM supplements significantly increased the average daily feed intake (ADFI) of pigs by approximately 10% and 9% (*p* = 0.038), respectively, compared to the control group. The KM50 group showed a trend towards increased feed intake to weight gain ratio (F/G) in the initial period (*p* = 0.058). However, no significant effects were observed on final weight (FW), average daily weight gain (ADG), ADFI, or F/G during Periods 2 and 3, as well as the entire experimental duration (*p* > 0.05).

**Figure 1:**
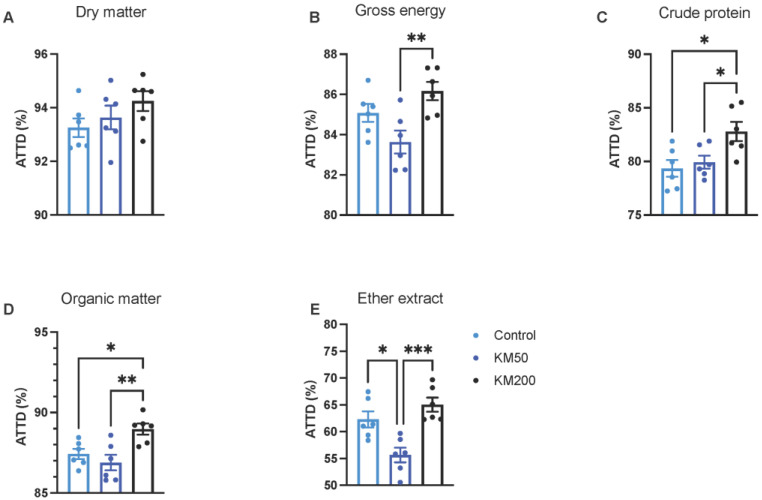
Impact of different KM doses on the apparent total tract digestibility of pigs. (**A**) Apparent total tract digestibility (ATTD) of dry matter; (**B**) ATTD of gross energy; (**C**) ATTD of crude protein; (**D**) ATTD of organic matter; (**E**) ATTD of ether extract. Control: Basal diet; KM50: 50 mg/kg KM, subtherapeutic KM diet; KM200: 200 mg/kg KM, therapeutic KM diet; KM: Kitasamycin. All values are presented as means ± standard error (*n* = 6). * *p* < 0.05; ** *p* < 0.01; *** *p* < 0.001 using one–way ANOVA analysis with Tukey multiple comparisons tests for gross energy, crude protein, organic matter, and ether extract; and the Kruskal–Wallis test with post hoc Dunn’s multiple comparisons test for dry matter, respectively.

To explore the impact of KM on feed intake utilization, the ATTD of nutrients was assessed. In comparison to the control group, the KM50 group displayed no discernible differences in ATTD for dry matter (Figure 1A), gross energy (Figure 1B), crude protein (Figure 1C), and organic matter (Figure 1D). However, the KM50 group exhibited a significant reduction in the ATTD of ether extract (Figure 1E) (*p* = 0.011) compared to the control group. The KM200 group demonstrated elevated ATTD for crude protein (*p* = 0.028) and organic matter (*p* = 0.049) compared to the control group. Notably, the KM50 group exhibited lower ATTD for gross energy, crude protein, organic matter, and ether extract in contrast to the KM200 group (*p* < 0.05).

### 3.2. Subtherapeutic KM Supplementation Enhanced Back Fat Thickness and Fat Content in the LM

**Figure 2:**
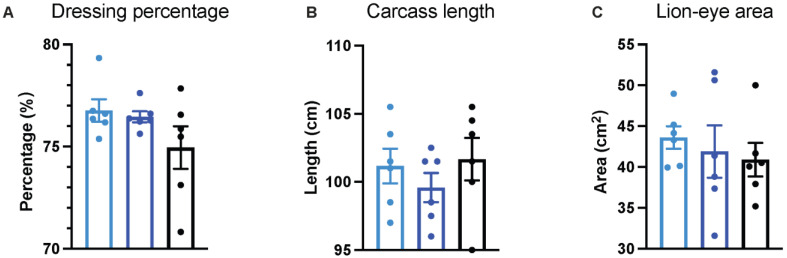

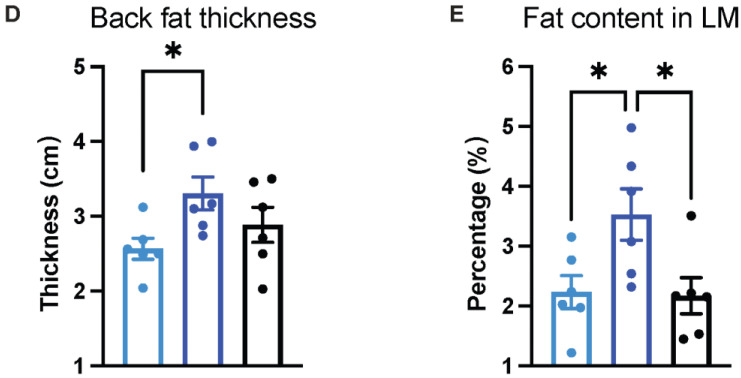
Impact of different KM doses on carcass traits of pigs. (**A**) Dressing percentage; (**B**) carcass length; (**C**) lion—eye area; (**D**) back—fat thickness; (**E**) fat content in the LM. Control: Basal diet; KM50: 50 mg/kg KM, subtherapeutic KM diet; KM200: 200 mg/kg KM, therapeutic KM diet. KM: Kitasamycin; LM: Longissimus dorsi muscle. All data are presented as mean ± standard error (*n* = 6). * *p* < 0.05 using one–way ANOVA with Tukey’s multiple comparisons test for carcass length, lion—eye area, back fat thickness, and fat content in the longissimus dorsi muscle, and the Kruskal–Wallis test with post hoc Dunn’s multiple comparisons test for dressing percentage, respectively.

The carcass trail was conducted to investigate the impact of different KM doses on fat accumulation. No noticeable differences were observed in dressing percentage (Figure 2A), carcass length (Figure 2B), or lion—eye area (Figure 2B) (*p* > 0.05). However, the KM50 group exhibited a significant elevation in both average back fat thickness (Figure 2D, *p* = 0.018) and fat content in the LM (Figure 2E, *p* = 0.042) compared to the control diet. Additionally, there were no substantial differences in back—fat thickness and fat content in the LM between the control group and the KM200 group (*p* > 0.05).

### 3.3. Subtherapeutic KM Supplementation Elevated Gene Expression Relating to Lipogenesis and Inflammatory Factors in the LM

To delve into the mechanistic aspects of the impact of different KM doses on fat accumulation in the LM, this study assessed the gene expression associated with lipid synthesis, lipolysis, and inflammatory factors in the LM.

**Figure 3:**
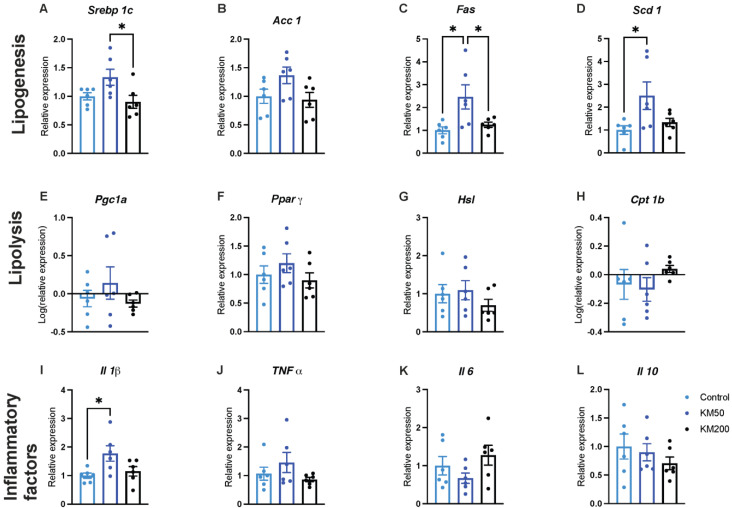
Impact of different KM doses on gene expression related to lipid synthesis, lipolysis, and inflammatory factors in the LM of pigs. Gene expression levels of *Srebp1c* (**A**), *Acc1* (**B**), *Fas* (**C**), *Scd1* (**D**), *Pgc 1α* (**E**), *Ppar γ* (**F**), *Hsl* (**G**), *Cpt 1b* (**H**), *Il 1β* (**I**), *TNF α* (**J**), *Il 6* (**K**), and *Il 10* (**L**) were assessed through qPCR. Control: Basal diet; KM50: 50 mg/kg KM, subtherapeutic KM diet; KM200: 200 mg/kg KM, therapeutic KM diet. *Acc*: Acetyl–CoA carboxylase; *Cpt*: Carnitine palmitoyl transferase; *Fas*: Fatty acidsynthase; *Hsl*: Hormone–sensitive lipase; *Il*: Interleukin; KM: Kitasamycin; *Pgc 1α*: Peroxisome proliferator–activated receptor–*γ* coactivator 1α; *Ppar γ*: Peroxisome proliferator–activated receptor *γ*; *Scd*: Stearoyl–coenzyme A desaturase; *Srebp1c*: Sterol regulatory element–binding protein 1c; *TNF α*: Tumor necrosis factor α. All data are shown as mean ± standard error (*n* = 6). * *p* < 0.05 using one–way ANOVA with Tukey’s multiple comparisons test for gene expression of *Srebp1c*, *Acc1*, *Fas*, *Scd1*, *Ppar γ*, *Hsl*, *Il 1β*, *TNF α*, *Il 6*, and *Il 10*, and the logarithmically transformed data of gene expression of *Pgc 1α* and *Cpt 1a*.

In comparison to the control group, the KM50 diet increased the expression of genes associated with lipogenesis including sterol regulatory element binding protein-1c (*Srebp 1c*, Figure 3A, *p* = 0.117), acetyl—CoA carboxylase 1 (*Acc1*, Figure 3B, *p* = 0.159), fatty acid synthase (*Fas*, Figure 3C, *p* = 0.016), and stearoyl—coenzyme A desaturase (*Scd1*, Figure 3D, *p* = 0.034) by 33%, 37%, 146%, and 150%, respectively. Interestingly, no significant differences were observed in gene expression of these lipogenic factors between the control group and the KM200 group (*p* > 0.05). However, the KM50 group exhibited higher gene expression than the KM200 group (*p* < 0.05), aligning with the increased fat content in the LM (Figure 2E), indicating KM50 diet—induced fat accumulation may be via upregulating gene expression of lipid synthesis in the LM.

No significant differences were observed in the expression of genes relating to lipolysis including peroxisome proliferator-activated receptor *γ* (*Ppar γ*, Figure 3F), and hormone-sensitive lipase (*Hsl*, Figure 3G), log (peroxisome proliferator-activated receptor-γ coactivator (*Pgc*) 1α gene expression) (Figure 3E), or log (carnitine palmitoyl transferase (*Cpt*) 1b gene expression) (Figure 3H) (*p* > 0.05) among the three groups, suggesting no impact of KM on gene expression associated with lipolysis.

Pigs fed the KM50 diet exhibited higher gene expression of *Il 1β* (Figure 3I, *p* = 0.029) compared to the control group. The gene expression of *TNF α* (Figure 3J), *Il 6* (Figure 3K), and *Il 10* (Figure 3L) were not altered by different doses of KM (*p* > 0.05).

### 3.4. Subtherapeutic KM Supplementation Elevated Plasma Total Cholesterol

**Figure 4:**
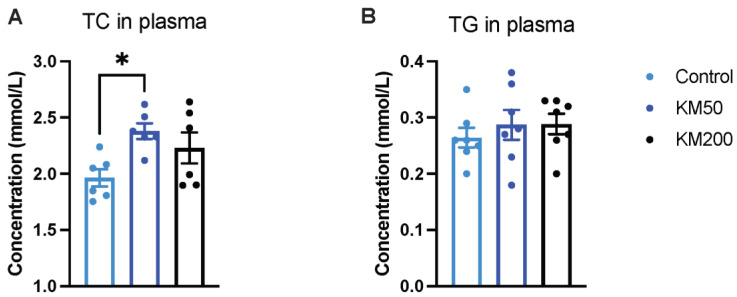
Impact of different KM doses on TC and TG concentrations in plasma. (**A**) TC concentration in plasma; (**B**) TG concentration in plasma. Control: Basal diet; KM50: 50 mg/kg KM, subtherapeutic KM diet; KM200: 200 mg/kg KM, therapeutic KM diet. KM: Kitasamycin; TC: Total cholesterol; TG: Total triglyceride. All data are shown as mean ± standard error (*n* = 6). * *p* < 0.05 using one–way ANOVA with Tukey’s multiple comparisons test.

To further explore the underlying mechanisms of different doses of KM on fat accumulation in the LM, plasma concentrations of TC and TG were assessed to explore potential crosstalk between plasma and LM. Pigs exposed to KM50 exhibited an elevated concentration of plasma TC (Figure 4A, *p* = 0.026) compared to those fed the control diet. However, no significant differences in TC concentration were observed between the control group and the KM200 group (*p* > 0.05). The data on TC concentrations in plasma align with the increased content (Figure 2E) and lipogenic gene expression (Figure 3B–D) in the LM in the KM50 group, suggesting a dose—related effect of KM on plasma TC concentrations. Dietary KM supplementation had no discernible effect on the concentration of plasma TG (Figure 4B, *p* > 0.05).

### 3.5. The Impact of Different Doses of KM on Intestinal Index, Cecal Microflora, SCFAs and Their Receptors

This study further investigated the effects of KM on the intestinal index, cecal microflora, and SCFAs.

**Figure 5:**
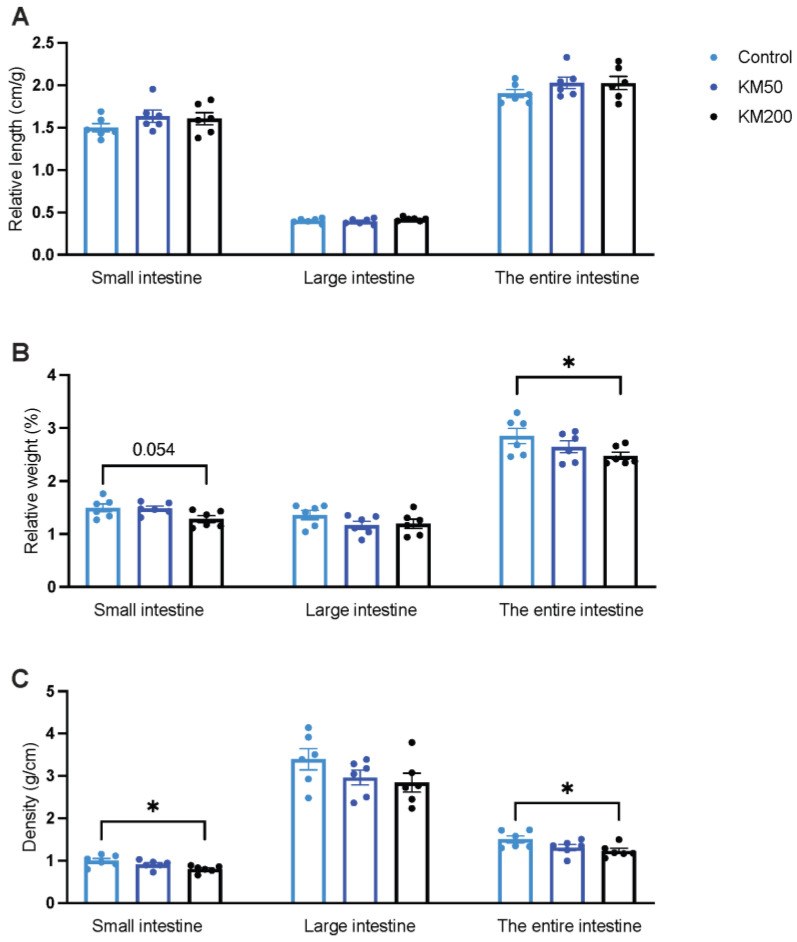
Impact of different KM doses on the intestinal index of pigs. This figure shows the impact of different KM doses on the relative length (**A**), relative weight (**B**) and relative density (**C**) of the small intestine, large intestine, and whole intestine. Control: Basal diet; KM50: 50 mg/kg KM, subtherapeutic KM diet; KM200: 200 mg/kg KM, therapeutic KM diet. All data are presented as mean ± standard error (*n* = 6). * *p* < 0.05 using one–way ANOVA with Tukey’s multiple comparisons tests.

Parameters such as relative length, relative weight and density of the intestinal tract serve as indicators of intestinal absorption capacity. No discernible changes were observed in the relative length of the small intestine, large intestine, and whole intestine among the groups (Figure 5A, *p* > 0.05). Pigs fed KM200 diets displayed a reduction in the relative weight (Figure 5B) of both the small intestine and the entire intestine by 14% (*p* = 0.054) and 13% (*p* = 0.032), respectively. Additionally, the KM200 diet resulted in a decreased density (Figure 5C) of the small intestine and the entire intestine compared to the control group (*p* < 0.05). These findings collectively indicate a dose-related effect of KM on the intestinal index.

**Figure 6:**
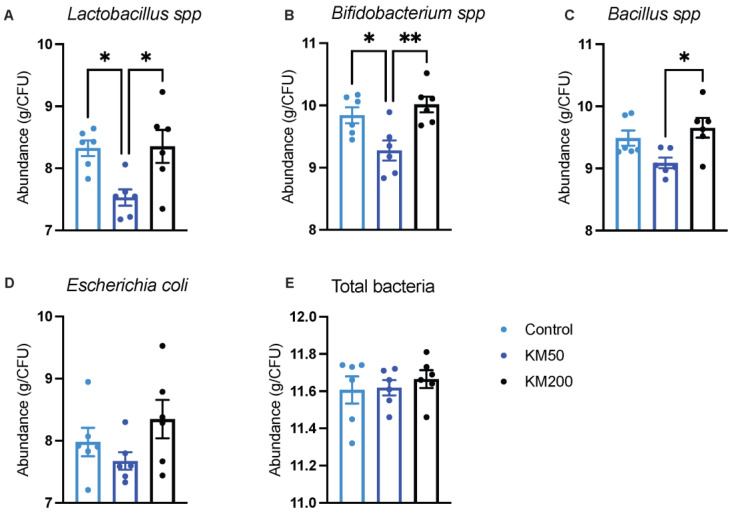
Impact of different KM doses on the abundance of cecal microflora in pigs (lg(copies/g)). The abundance of *Lactobacillus* spp. (**A**), *Bifidobacterium* spp. (**B**), *Bacillus* spp. (**C**), *Escherichia coli* spp. (**D**), and total bacteria (**E**) was analysed using qPCR. Control: Basal diet; KM: Kitasamycin; KM50: 50 mg/kg KM, subtherapeutic KM diet; KM200: 200 mg/kg KM, therapeutic KM diet. All data are presented as mean ± standard error (*n* = 6). * *p* < 0.05; ** *p* < 0.01 using one–way ANOVA with Tukey’s multiple comparisons test for the abundance of *Lactobacillus* spp., *Bifidobacterium* spp., *Escherichia coli* spp., and total bacteria, and the Kruskal–Wallis test with the post hoc Dunn’s multiple comparisons test for the abundance of *Bacillus* spp., respectively.

The KM50 diet led to a reduction of approximately 10% in the abundance of *Lactobacillus* spp. (Figure 6A, *p* = 0.022), 6% in the abundance of *Bifidobacterium* spp. (Figure 6B, *p* = 0.028), 4% in the abundance of *Bacillus* spp. (Figure 6C, *p* = 0.095), and 4% in the abundance of *Escherichia coli* (Figure 6D, *p* = 0.117) in the cecum compared to the control group. These data suggest a dose-related effect of KM on the abundance of microflora in the cecum. Dietary KM supplementation did not affect the abundance of total bacteria (Figure 6E) in the cecum (*p* > 0.05).

**Figure 7:**
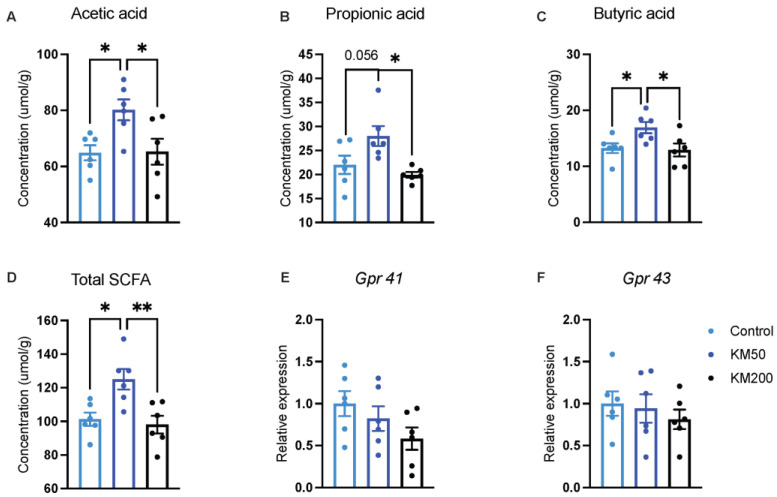
Impact of different KM doses on concentrations of SCFAs and gene expression of their receptors in the cecum of pigs. The concentrations of acetic acid (**A**), propionic acid (**B**), butyric acid (**C**), and total SCFAs (**D**) were determined using a gas chromatographic system. Gene expression of SCFA receptors, *Gpr41* (**E**) and *Gpr43* (**F**), was measured through qPCR. Control: Basal diet; KM50: 50 mg/kg KM, subtherapeutic KM diet; KM200: 200 mg/kg KM, therapeutic KM diet. *Gpr*: G protein—coupled receptor; KM: Kitasamycin; SCFAs: short–chain fatty acids. All data are presented as mean ± standard error (*n* = 6). * *p* < 0.05, ** *p* < 0.01 using one–way ANOVA with Tukey’s multiple comparisons test.

The KM50 group demonstrated heightened concentrations of acetic acid (Figure 7A), butyric acid (Figure 7C), and total SCFAs (Figure 7D) in the cecum compared to the control group and the therapeutic KM group (*p* < 0.05). These increased concentrations suggest a dose-related effect of KM on SCFA concentrations. Moreover, the KM50 group showed increased concentrations of propionic acid (Figure 7B) compared to the control (*p* = 0.056) and KM200 (*p* = 0.010) groups. Simultaneously, no discernible differences were observed in the gene expression of SCFA receptors, including *Gpr 41* (Figure 7E) and *Gpr 43* (Figure 7F), among these groups (*p* > 0.05).

## 4. Discussion

This study delves into the impact of KM, a macrolide antibiotic mainly targeting G—positive bacteria, on intramuscular lipid metabolism, intestinal indices, and cecal microflora and its metabolic products in pigs. Key findings from this investigation are as follows: (1) both subtherapeutic and therapeutic KM diets promoted ADFI in Period 1, as well as ADG and ADFI, albeit not statistically significant throughout the entire process; (2) Dose—dependent effects of KM on fat deposition in the LM were observed, accompanied by modulation of lipogenic and inflammatory gene expression, alternations in intestinal weight and density, and changes in cecal microflora and SCFAs. (3) Therapeutic KM diet, compared to subtherapeutic KM diets, led to increased ATTD of nutrients and the abundance of some cecal microbiota, but decreased fat content in the LM, along with decreased expression of lipogenic genes and concentrations of SCFAs in the cecum. These findings collectively highlight the adverse effects of different doses of KM on intramuscular fat deposition and cecal microbiota and SCFAs in pigs, providing valuable insights into the potential implications and mechanisms of KM utilization in pig husbandry.

While previous research has underscored the growth-promoting effects of certain antibiotics in animals, the impact of KM on growth performance in pigs has remained relatively unexplored. This study revealed that both subtherapeutic and therapeutic KM diets enhanced ADFI initially. Notably, KM 50 and KM200 diets resulted in a 3.2% and 2.9% increase in FW, a 6.8% and 6.2% increase in ADG, and a 9.3% and 6.2% increase in ADFI, respectively. These findings align with observations from previous studies demonstrating sustained weight gain associated with antibiotics in various species [25,26]. However, conflicting results, as reported by Zhao et al. (2020), showed no influence on the growth performance of growing pigs with long-term diets with 50 mg/kg kitasamycin [27]. These conflicting findings may be attributed to animal age and the duration of antibiotic usage. The present study suggests that KM supplementation initially promoted growth in growing–finishing pigs with prolonged administration.

Interestingly, this study also observed a 10.7% decrease in the ATTD of ether extract following subtherapeutic KM treatment. In contrast to our findings, previous swine studies have reported that antibiotics can increase the ATTD of dry matter, crude protein, and gross energy, with no impact on the ATTD of ether extract due to enhanced nutrient retention [28,29,30]. The disparities in results may be attributed to differences in animal growth stages, the types of antibiotics used, and the composition of fat and protein in their diets. In this study, the decreased ATTD of ether extract may be associated with reduced activity of digestive and absorptive enzymes or compromised intestinal barrier function [14]. However, the precise mechanism underlying the decreased ATTD of ether extract induced by subtherapeutic KM requires further investigation.

In this study, pigs weighing approximately 60 kg, a critical period for fat accumulation, were selected. Considering that it typically takes around 8 weeks before reaching the industry harvest weight, this experiment was conducted for 8 weeks to investigate the effects of different doses of KM on lipid deposition in finishing pigs. The impact of antibiotics on lipid metabolism has drawn considerable attention, and this study contributes insights into the underlying mechanisms. Previous human studies have demonstrated that ectopic lipid accumulation in muscle serves as an indicator of insulin resistance and Type 2 diabetes [31,32]. The findings in this study align with this, as subtherapeutic KM treatment resulted in fat accumulation in back—fat thickness, the LM and plasma, highlighting the detrimental effects of prolonged subtherapeutic KM usage on ectopic fat deposition. Consistent with previous findings, subtherapeutic antibiotic doses have been associated with increased fat mass [10]. Lipid metabolism involves intricate processes regulated by numerous enzymes. Key transcription factors such as *Srebp1c* coordinate the transcription of genes crucial for lipid synthesis, including *Acc1* and *Fas* [33], crucial in regulating the synthesis of malonyl—CoA and palmitate, respectively [34,35]. Palmitate is subsequently converted into palmitoleate regulated by *Scd1*. This study observed elevated expression of lipogenic genes such as *Acc1*, *Fas* and *Scd1* in the LM following subtherapeutic KM treatment, suggesting that subtherapeutic KM treatment may induce lipid accumulation by increasing lipogenic gene expression in the LM. Additionally, regulatory elements like *Pgc1α* and *Ppars* are involved in FA lipolysis [36,37], while *Hsl* [38] and *Cpt* [39] play key roles in lipid hydrolysis and *β*-oxidation, respectively. Interestingly, this study did not observe changes in the expression of transcription factors and genes associated with lipolysis and *β*-oxidation. Furthermore, the study identified the upregulation of *Il1β*, which is linked with lipid metabolism by regulating insulin levels and lipase activity [40]. Notably, no such effects were observed in the therapeutic group, suggesting a dose—related effect of KM on fat accumulation. Thus, this study indicates a dose—dependent effect of KM on fat accumulation by promoting lipogenesis, coupled with the upregulation of *Il1β* in the LM.

The intestinal microflora and their metabolites play a pivotal role in modulating host lipid metabolism through direct and/or indirect mechanisms [18]. Antibiotic usage has been shown to disrupt the intestinal microbiota, impacting intestinal function [41]. This study investigated alternations in microbiota and their products to understand their connection with lipid deposition. This study revealed that the subtherapeutic KM diet decreased the abundance of *Lactobacillus* spp., *Bifidobacterium* spp. and *Bacillus* spp. while increasing the concentrations of acetic acid, propionic acid, and total SCFAs. SCFAs, being the principal end products of microbiota [42], can be absorbed as energy sources, actively contributing to lipid synthesis [43,44] and altering host metabolic efficiency [45], potentially leading to lipid ectopic deposition in other organs. This aligns with previous research associating subtherapeutic antibiotic doses with increased SCFAs and fat mass [10]. Additionally, previous studies have reported that alternations in microbiota and SCFAs could stimulate hepatic lipid synthesis [46,47] and intestinal barrier function [48]. 

This discrepancy in microflora and SCFAs observed in the KM50 group of this study could be attributed to specific microflora species responsible for SCFA production. For example, acetate is mainly produced by Bifidobacterium, propionate producers are Bacteroides species and Negativicutes, and butyrate tends to be produced by Firmicutes [49,50]. Interestingly, while the therapeutic KM diet had no effects on cecal bacteria and SCFAs, it decreased intestinal density while increasing intestinal length and ATTD of nutrients, consistent with the increase in ADG and ADFI over the entire period. These findings collectively suggest that KM200—induced ADG may be attributed to increased ATTD of nutrients through intestinal extraction. Intestinal microflora may play a beneficial role in utilizing food to synthesize and store fat by modulating digestion and absorption [51]. However, potential explanations for the lack of observed alterations in fat accumulation, gut microbiota, and SCFAs in the therapeutic KM group may include the development of bacterial resistance and its association with reduced intestinal density, impacting apparent fat digestibility. In vitro study has shown that prolonged KM usage can induce KM resistance [52]. Therefore, the unexpected lack of alternations in fat accumulation, gut microbiota and SCFAs in the therapeutic KM group may be attributed to prolonged exposure to a high dose of KM. 

Furthermore, this study highlighted a higher residual concentration of KM in the liver of the therapeutic group (35.70 ug/kg) compared to the subtherapeutic group (4.55 ug/kg). Moreover, a considerable amount of KM (approximately 23,176 ug of KM per kg of feces) was expelled into the environment by the therapeutic group. (Only a few samples determined the concentration of KM by High-Performance Liquid Chromatography, and the data were shown in Table S1) KM residue and excretion from pigs potentially contributed to bacteria resistance and posed environmental risks [46]. Future investigations into bacterial composition, diversity, and richness in the cecum are warranted to further elucidate the different impacts of nontherapeutic and therapeutic diets. Understanding the effects of KM on pig health and residue is crucial for farmers, and policymakers. This study provides evidence supporting the reduction in KM in humans and agriculture.

## 5. Conclusions

The study findings suggested that KM exhibits dose-related effects on lipid deposition, accompanied by changes in gene expression related to lipid metabolism in the LM, cecal microflora and SCFAs of pigs. Subtherapeutic KM administration resulted in fat accumulation in the LM, potentially linked to alternations in cecal microbiota and SCFAs. Conversely, therapeutic KM administration improved nutrient ATTD, possibly due to reduced intestinal density leading to enhanced nutrient absorption. These findings provide valuable insights into the multifaceted implications and mechanisms of KM utilization in pigs, highlighting the need for cautious administration to mitigate adverse effects on health and the environment.

## Figures and Tables

**Table 1:** Ingredients and nutrient compositions of basal diet, air dry basis %.

Ingredients	Content, %			Nutrient Compositions	Level ^c^, %		
	50–75 kg	75–100 kg	100–120 kg		50–75 kg	75–100 kg	100–120 kg
Maize (Crude protein 7.8%)	76.8	79.37	80.82	Digestible energy/(MJ/kg)	14.23	14.23	14.23
Soybean meal (Crude protein 43%)	19	17	16	Crude protein	14.59	14.06	13.62
Soybean oil	1.5	1.3	1.3	Ca	0.57	0.52	0.46
L-lysine·HCl	0.34	0.25	0.12	Total protein	0.47	0.43	0.41
DL-Methionine	0.06	0.04	0	Available protein	0.27	0.24	0.22
Threonine	0.1	0.06	0.01	Digestible lysine	0.85	0.73	0.61
Choline chloride	0.15	0.1	0.1	Digestible methionine	0.26	0.24	0.2
CaCO_3_	0.55	0.54	0.47	Digestible Threonine	0.52	0.46	0.4
CaHPO_4_	0.82	0.66	0.5	Digestible Tryptophan	0.13	0.12	0.12
Mineral Complex ^a^	0.3	0.3	0.3				
Vitamin Complex ^b^	0.03	0.03	0.03				
NaCl	0.35	0.35	0.35				
Total	100	100	100				

^a^ The premix provides the following per kg diet: Fe (FeSO_4_·H_2_O) 40.0 mg, Cu (CuSO_4_·5H_2_O) 3.0 mg, Mn (MnSO_4_·H_2_O) 2.0 mg, Zn (ZnSO_4_·H_2_O) 50.0 mg, I (KI) 0.14 mg, Se (Na_2_SeO_3_) 0.15 mg. ^b^ The vitamin premix provides following per kg diet: VA 12000 IU, VD_3_ 3000.0 IU, VE 7.5 IU, VK_3_ 1.5 mg, VB_1_ 0.6 mg, VB_2_ 4.8 mg, VB_6_ 1.8 mg, VB_12_ 9 ug, folic acid 0.15 mg, nicotinic acid 1.05 mg. ^c^ Nutrient levels were calculated values.

**Table 2:** Specific primer sequences and probes for real—time qPCR.

Items	Primer/Probe Name and Sequence (5′–3′)	Product Length/bp
Total bacteria	F, ACTCCTACGGGAGGCAGCAG	
	R, ATTACCGCGGCTGCTGG	200
*Lactobacillus* spp.	F, GAGGCAGCAGTAGGGAATCTTC	
	R, CAACAGTTACTCTGACACCCGTTCTTC	126
	P, (FMA)AAGAAGGGTTTCGGCTCGTAAAACTCTGTT(BHQ-1)	
*Bifidobacterium* spp.	F, CGCGTCCGGTGTGAAAG	
	R, CTTCCCGATATCTACACATTCCA	121
	P, (FMA) ATTCCACCGTTACACCGGGAA(BHQ-1)	
*Bacillus* spp.	F, GCAACGAGCGCAACCCTTGA	
	R, TCATCCCCACCTTCCTCCGGT	92
	P, (FMA)CGGTTTGTCACCGGCAGTCACCT(BHQ-1)	
*Escherichia-coli*	F, CATGCCGCGTGTATGAAGAA	
	R, CGGGTAACGTCAATGAGCAAA	96
	P, (FMA)AGGTATTAACTTTACTCCCTTCCTC(BHQ-1)	

F: Forward primer; R: Reverse primer; P: Probe.

**Table 3:** Primer sequences and annealing temperature.

Target Gene	Forward Primer 5′−3′	Reverse Primer 5′−3′	Accession Number
Lipid metabolism factors			
*Srebp 1c*	GCGACGGTGCCTCTGGTAGT	CGCAAGACGGCGGATTTA	NM_214157.1
*Acc 1*	AGCAAGGTCGAGACCGAAAG	TAAGACCACCGGCGGATAGA	NM_001114269
*Fas*	CTACGAGGCCATTGTGGACG	AGCCTATCATGCTGTAGCCC	NM_001099930
*Scd 1*	CCACTATGACCCGGAAGACG	TTGAACGCGATGAGGGTGAA	NM_001007191.1
*Pgc 1α*	CCCGAAACAGTAGCAGAGACAAG	CTGGGGTCAGAGGAAGAGATAAAG	NM-213963
*Ppar γ*	CCAGCATTTCCACTCCACACTA	GACACAGGCTCCACTTTGATG	NM_214379.1
*Hsl*	CACAAGGGCTGCTTCTACGG	AAGCGGCCACTGGTGAAGAG	NM_214315
*Cpt 1b*	CCACTATGACCCGGAAGACG	TTGAACGCGATGAGGGTGAA	NM_214293
Inflammatory factors			
*Il 1β*	ACGTGCAATGATGACTTTGTCTG	AGAGCCTTCAGCATGTGTGG	NM_214055.1
*Il 6*	TTCACCTCTCCGGACAAAAC	TCTGCCAGTACCTCCTTGCT	NC_010451.3
*Il 10*	TAATGCCGAAGGCAGAGAGT	GGCCTTGCTCTTGTTTTCAC	NM_214041.1
*TNFα*	ACCACGCTCTTCTGCCT	CACTGTCACCTGGAAGCAGAG	NM_214022.1
SCFA receptors			
*Gpr 43*	TCATGGGTTTCGGCTTCTACAG	GTACTGAACGATGAACACGACG	EU_122439.1
*Gpr 41*	ACTACTTCTCATCCTCGGGGTT	CTCCACTTCGCTCTTCTTCAGT	JX_566879.1
*β actin*	TCTGGCACCACACCTTCT	TGATCTGGGTCATCTTCTCAC	DQ_178122

*Acc*: Acetyl-CoA carboxylase; *Cpt*: Carnitine palmitoyl transferase; *Fas*: Fatty acid synthase; *Gpr*: G protein-coupled receptor; *Hsl*: Hormone-sensitive lipase; *Il*: Interleukin; *Pgc 1α*: Peroxisome proliferator-activated receptor-γ coactivator 1α; *Ppar γ*: Peroxisome proliferator-activated receptor γ; *Scd*: Stearoyl-coenzyme A desaturase; *Srebp*: Sterol regulatory element-binding protein; *TNF α*: Tumor necrosis factor α.

**Table 4:** Impact of different doses of KM on the growth performance of pigs.

Item	Control	50 mg/kg KM	200 mg/kg KM	*p*-Value
Period 1				
Initial BW (kg)	63.32 ± 1.00	63.32 ± 1.00	63.32 ± 1.01	1
FW (kg)	78.34 ± 1.27	78.11 ± 1.25	79.21 ± 1.09	0.795
ADG (g)	1072.96 ± 25.55	1094.05 ± 35.99	1135.20 ± 24.50	0.153
ADFI (g)	2797.13 ± 62.63 ^a^	3084.54 ± 72.72 ^b^	3040.39 ± 83.75 ^b^	0.038
F/G	2.61 ± 0.07	2.82 ± 0.06	2.68 ± 0.05	0.058
Period 2				
FW (kg)	96.75 ± 2.07	98.96 ± 1.95	99.50 ± 2.00	0.599
ADG (g)	876.53 ± 54.09	992.86 ± 44.81	945.13 ± 49.27	0.247
ADFI (g)	2892.91 ± 140.38	3189.69 ± 213.76	3233.28 ± 132.66	0.529
F/G	3.32 ± 0.10	3.22 ± 0.18	3.36 ± 0.06	0.727
Period 3				
FW (kg)	119.82 ± 1.81	123.64 ± 3.66	123.32 ± 2.64	0.576
ADG (g)	823.93 ± 39.20	941.72 ± 58.02	850.71 ± 33.90	0.18
ADFI (g)	2998.72 ± 70.56	3288.04 ± 131.85	3028.28 ± 118.02	0.152
F/G	3.69 ± 0.20	3.52 ± 0.22	3.57 ± 0.08	0.784
The whole process				
ADG (g)	896.82 ± 13.86	957.46 ± 31.95	952.38 ± 32.09	0.152
ADFI (g)	2917.63 ± 75.25	3189.67 ± 148.77	3099.37 ± 109.59	0.261
F/G	3.25 ± 0.07	3.32 ± 0.11	3.25 ± 0.08	0.742

Control: Basal diet; KM50: 50 mg/kg KM, subtherapeutic KM diet; KM200: 200 mg/kg KM, therapeutic KM diet. ADFI: Average daily feed intake; ADG: Average daily weight gain; BW: Body weight; F/G: The ratio of feed to weight gain; FW: Final weight; KM: Kitasamycin. All values are expressed as means ± standard error (*n* = 14). ^a,b^ Rows with different superscript letters indicate significant differences using one–way ANOVA analysis with Tukey’s multiple comparisons test (*p* < 0.05).

## Data Availability

All data included in this paper are available upon request by contact with the corresponding authors.

## Supplementary Materials

The following supporting information can be downloaded at: https://www.mdpi.com/article/10.3390/ani14071057/s1, Table S1. Kitasamycin concentrations in different samples.
