# Supplementary material for: Subtherapeutic Kitasamycin Promoted Fat Accumulation in the Longissimus Dorsi Muscle in Growing–Finishing Pigs
Source: Animals (Basel). 2024 Mar 30;14(7):1057. doi: 10.3390/ani14071057 (PMC11010921; doi:10.3390/ani14071057)
Supplement: Supplementary file 1 [file animals-14-01057-s001.zip › animals-2896157-supplementary.pdf]

Supplemental Materials

Table S1. Kitasamycin concentrations in different samples

samples	Treatment	KM concentrations (ug/kg)
	Control	Undetected (<1.0)
Liver	KM50	4.55
	KM200	35.7
Faeces	KM200	23176
LM	KM200	Undetected (<1.0)
Urine	KM200	Undetected (<1.0)

The KM concentrations in different samples were assessed utilizing LC-MC methods at the Kebiao Technology R&D Center in Qingdao, China. KM: kitasamycin; LM: longissimus dorsi muscle area.
